# Ultrasensitive amplicon barcoding for next-generation sequencing facilitating sequence error and amplification-bias correction

**DOI:** 10.1038/s41598-020-67290-1

**Published:** 2020-06-29

**Authors:** Ibrahim Ahmed, Felicia A. Tucci, Aure Aflalo, Kenneth G. C. Smith, Rachael J. M. Bashford-Rogers

**Affiliations:** 1 Department of Medicine, University of Cambridge, Cambridge, United Kingdom; 2 Present Address: Faculty of Biology, Medicine and Health, University of Manchester, Michael Smith Building, Oxford Road, Manchester, M13 9PT UK; 3 Wellcome Centre for Human Genetics, University of Oxford, Oxford, United Kingdom; 4 Department of Pathology, University of Cambridge, Cambridge, United Kingdom; 5 Cambridge Institute of Therapeutic Immunology and Infectious Disease, Jeffrey Cheah Biomedical Centre, University of Cambridge, Cambridge, United Kingdom

**Keywords:** Biotechnology, VDJ recombination, Genotype, Mutation, Sequencing

## Abstract

The ability to accurately characterize DNA variant proportions using PCR amplification is key to many genetic studies, including studying tumor heterogeneity, 16S microbiome, viral and immune receptor sequencing. We develop a novel generalizable ultrasensitive amplicon barcoding approach that significantly reduces the inflation/deflation of DNA variant proportions due to PCR amplification biases and sequencing errors. This method was applied to immune receptor sequencing, where it significantly improves the quality and estimation of diversity of the resulting library.

## Introduction

Amplicon sequencing is often the basis for characterizing DNA variant proportions, and is routinely used in many areas including tumor heterogeneity^1^, 16S microbiome^2^, viral^3^, CRISPR/Cas9 library screens^4^ and immune receptor sequencing^5^. However, the ability to accurately quantify the proportions of DNA variants is hampered by amplification biases that lead to inflation/deflation of some DNA amplicons, as well as the inability to correct sequencing errors (Fig. S1a). To overcome the amplification biases in DNA-based amplification and sequencing, we developed a novel generalizable ultrasensitive amplicon barcoding approach that significantly reduces the inflation/deflation of DNA variant proportions from PCR amplification biases and sequencing errors.

Amplification biases from RNA starting material have been largely addressed by the introduction of unique molecular identifiers (UMIs) in the reverse transcription primers (barcoded primers), thus subsequent PCR amplification of each cDNA molecule can be quantified and corrected through the capture of the UMI barcode. However, when starting from a DNA template, this approach cannot be used. Previous attempts at generating barcoded PCR amplicons from DNA using barcoded primers via standard exponential PCR amplification leads to the preferential amplification of PCR amplicons rather than template^6^ and thus resulting  in significant amplification biases (Fig. S1b).

**Figure 1:**
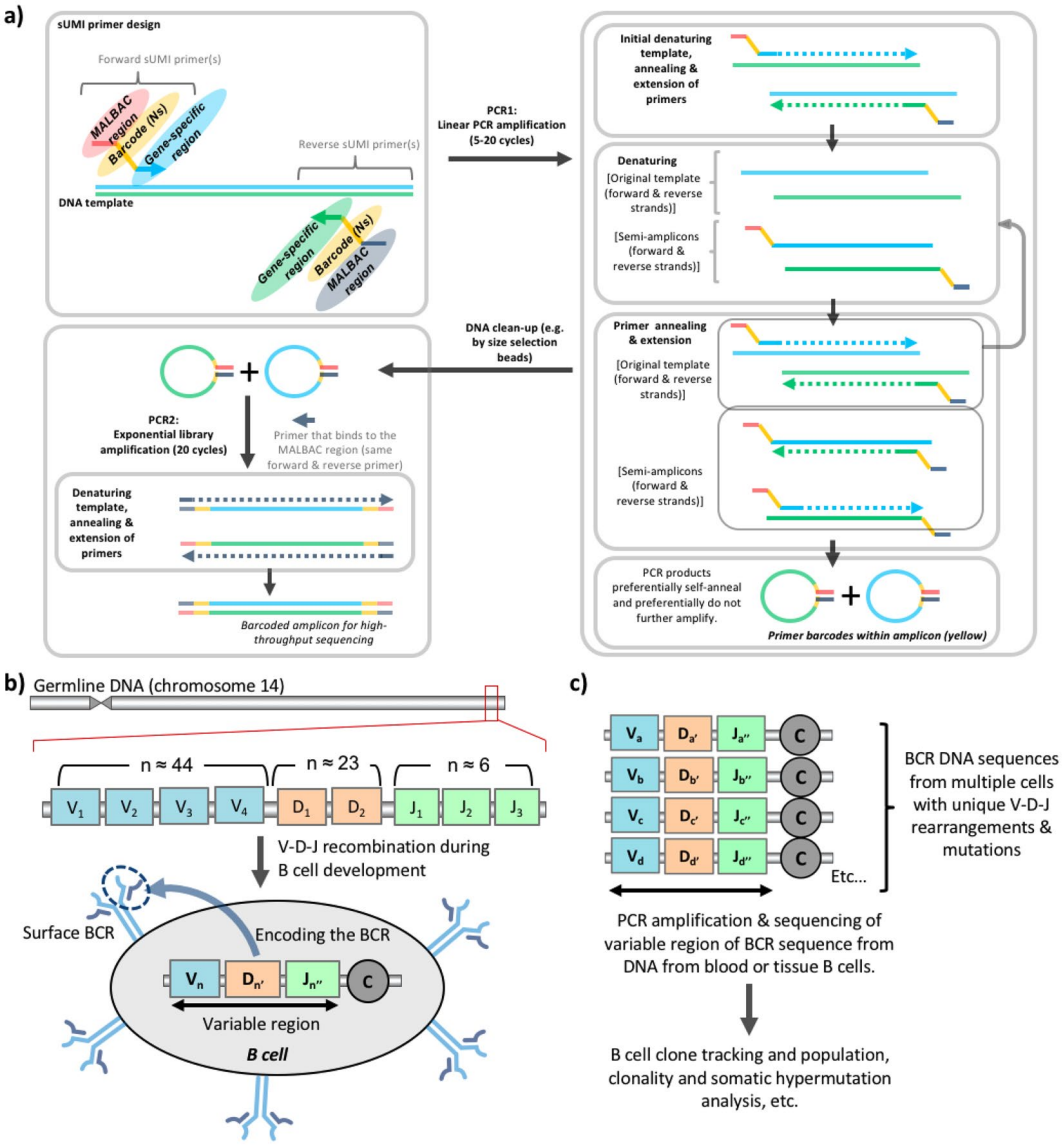
Schematic of the sUMI-seq PCR with DNA molecular barcoding approach. (**a**) Schematic diagram of sUMI-seq. Quantification of pools of DNA variants can be important across multiple fields in biology, including B cell receptor (BCR) repertoire sequencing. MALBAC-barcoded primer design is shown. Amplicons from PCR1, after a clean-up step, are amplified in PCR2 using forward primers priming gene specific regions (as in PCR1) and reverse universal primers binding to the MALBAC regions (**b**) Schematic diagram of BCR rearrangement: B cells are generated from haematopoietic stem cells. The IGH gene locus on chromosome 14 (in humans) encodes for multiple distinct copies of the variable (V), diversity (D), and joining (J) genes, with functional IGH BCR (one functional allele per cell) generated during B cell differentiation by site-specific V-D-J recombination. Random deletions and insertions of nucleotides during recombination results in sequence diversification at the gene junctional regions. Rearranged BCR genes can be further diversified through somatic hypermutation (SHM) upon B cell activation. (**c**) A BCR repertoire is defined as the BCRs collection from a B cell population, such as from blood or tissues. B cell DNA can be used to construct BCR libraries, using multiplex PCR with forward primers annealing to all the variable VH gene families (IgH V1–7) and reverse primers annealing to the JH gene families (IgH J1-6).

To overcome these issues, we established a sUMI-seq PCR amplification using barcoded primers that generate self-annealing amplicons (Fig. 1a, denoted sUMI-seq primers. Secondary structure-assisted UMI incorporation, amplification and sequencing). These sUMI-seq primers contain three key regions: (1) the target gene-specific region, (2) a UMI primer barcode (8 bp), and (3) a region based on multiple annealing and looping-based amplification cycles (MALBAC) methodology^7^, in which the PCR products are able to self-anneal forming MALBAC amplicon loops. These amplicon loops preferentially do not further amplify due to the thermodynamic and kinetic preference for loop closure compared to further primer annealing to the open and available original DNA template (Fig. S2). This will result in a close-to-linear amplification, rather than standard exponential amplification, of template DNA due to the unavailability of the MALBAC amplicon loops to further amplify. This first PCR (PCR1) is followed by a cleanup step to remove unbound primers and primer dimers. Then a second PCR (PCR2), with primers annealing to the common MALBAC region of PCR1 amplicons, generates linearized amplicons that are amenable for library preparation and high-throughput sequencing. A bioinformatics pipeline was developed to identify the primer barcodes, to correct for amplification frequency and to correct sequencing errors through alignment of sequences sharing the same barcode (code made available at https://github.com/rbr1/sUMI_processing_pipeline).

sUMI-seq is made possible by two key innovations: firstly, the PCR amplification step (PCR1) using the sUMI-seq primers allows for preferential amplification of the template DNA and minimal amplification of the MALBAC looped amplicons. Secondly, linearization of the self-annealed PCR amplicons in PCR 2 leads to increased sensitivity in the face of low template DNA input. The use of barcoded sample primers in PCR2 allows for sample pooling, and efficient library preparation and sequencing.

One area in which quantification of DNA variants is important is in B cell receptor sequencing. B cell receptors (BCRs) are membrane-bound immunoglobulins (Igs) which are secreted as antibodies by antibody secreting cells (plasma cells), which differentiate from naïve or memory B cells upon antigen activation. The huge diversity of the antibody repertoire is due to DNA recombination of variable gene segments (V, (D), J) at the Ig heavy (IgH) and light (IgL) chain loci during B cell ontogeny, and subsequent acquisition of somatic hypermutation (SHM) in activated B cells. Both IgH and IgL variable regions are further subdivided into four framework regions (FWR 1–4), determining the antibody folding, and into three complementarity determining regions (CDR 1–3), involved in antigen binding. BCRs represent unique markers for each B cell clone (Fig. 1b,c). The BCR repertoire analysis by BCR gene deep sequencing allows measurement of the diversity and complexity of B cell response, and to identify clonal related B cells (B cell clones) which correlate with different immunological conditions. BCR repertoire analysis from blood or tissues by high-throughput sequencing has been used to provide powerful insights into B cell biology and tracking B cell clones in the context of health^6,8^, autoimmunity^9^, cancer^10,11^, infection^12^, vaccination^13^ and in other diseases. BCR sequencing from RNA has been established using UMIs of each RNA molecule to accurately quantify relative BCR RNA frequencies^6^. Despite the successes of RNA-based BCR repertoire sequencing, RNA can be a sub-optimal substrate for BCR sequencing. The level of Ig transcripts is upregulated through B cell maturation and activation upon antigen encounter, with plasma cells having the highest amount of Ig mRNA per cell. This may lead to inflation or deflation of the detected B cell clonotypes of a repertoire. This was shown clearly in the detection of B cell acute lymphocytic leukaemia^10^, where lower numbers of BCR RNA molecules per leukemic cell compared to non-leukemic B cells lead to a significant underrepresentation of patient tumor proportion. This major limitation can be overcome through sequencing from DNA, as B cells carry one functional BCR allele per cell (one B cell – one antibody). However, no reliable method for molecular barcoding during PCR amplification of DNA has yet been established, thus leading to potential amplification biases in the resulting sequencing data.

Here we develop and validate a novel generalizable ultrasensitive amplicon barcoding approach and apply it to BCR sequencing, where it significantly improves the quality and estimation of diversity of the resulting library.

## Results and Discussion

**Figure 2:**
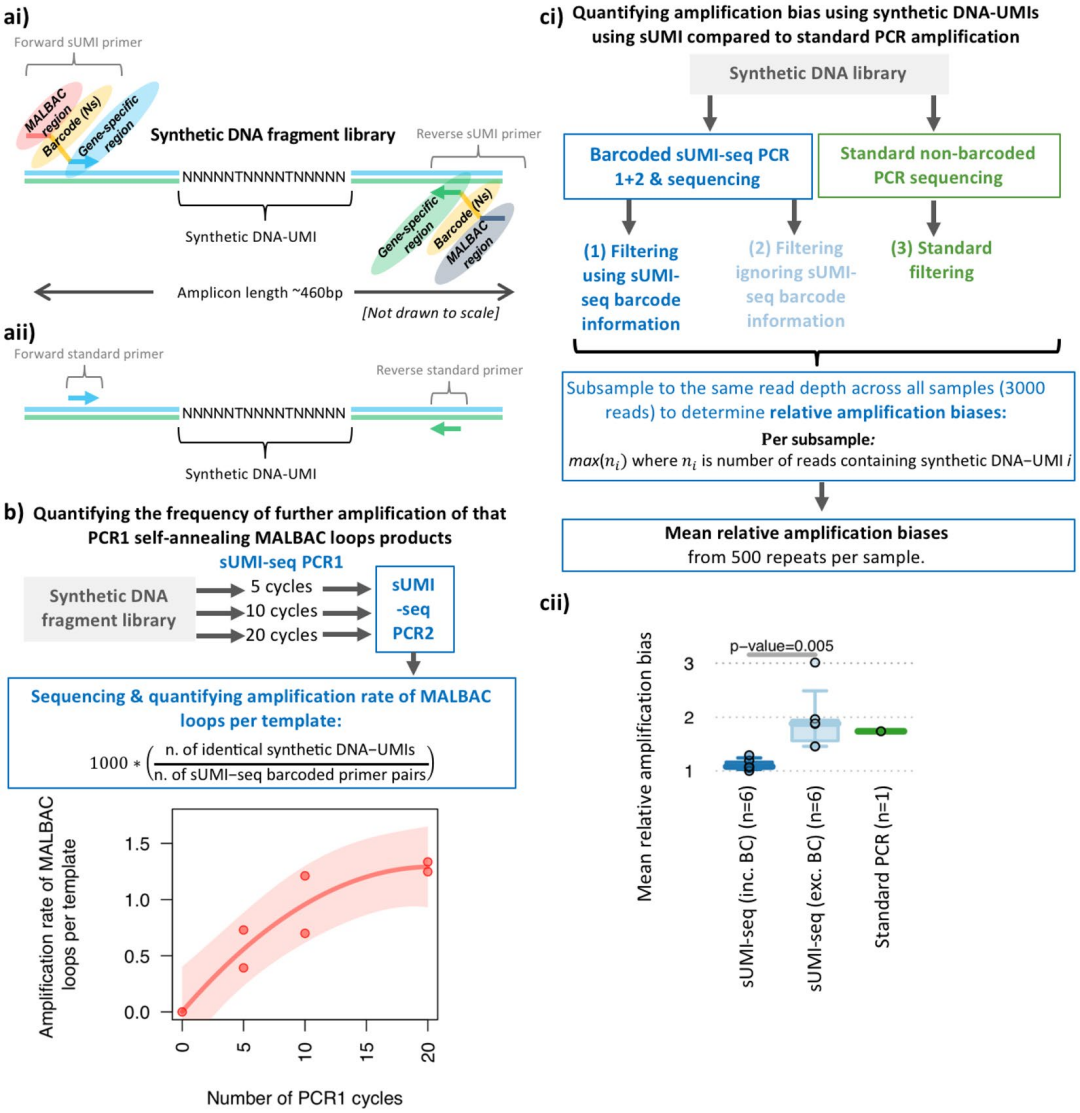
sUMI-seq more accurately quantified B cell receptor sequence repertoires. (**ai**) Schematic diagram of the synthetic barcoded DNA fragments (representing a BCR sequence) used to quantify the DNA capture and amplification biases of DNA sequencing methods. A library of synthetic DNA fragments was designed based on a BCR sequence and containing a region of random nucleotides such that each DNA fragment is unique (termed synthetic DNA-UMIs). These were amplified using the sUMI-seq primers (**ai,aii**) and standard PCR primers with gene specific sequences. (**b**) Quantifying the frequency of further amplification of that PCR1 self-annealing MALBAC loops products under 5, 10 and 20 PCR 1 cycles performed in duplicate. (**c**) Quantification of the amplification bias using synthetic DNA-UMIs and sUMI-seq compared to standard PCR amplification. (i) Schematic diagram of the experimental design and (ii) a boxplot of the mean relative amplification biases between sUMI-seq (filtering using barcode information), sUMI-seq (filtering ignoring barcode information), and standard PCR amplification without barcodes. The number of samples per box is provided in brackets. Wilcoxon tests were performed in R.

To test the effectiveness of sUMI-seq PCR, a synthetic DNA fragment library was designed containing an internal DNA barcode (referred to as synthetic DNA-UMI) unique to each DNA molecule (Figs. 2a, S3). This synthetic DNA fragment UMI library design was based on a BCR sequence. This means that both the synthetic DNA-UMI and BCR repertoire from clinical samples can be amplified using the same primer sets. Specifically, forward primers anneal to the IgH V genes (FR3) and reverse primers anneal to IgH J genes (Table S1), allowing the amplification of the IgH variable region encompassing the CDR3 which is the major determinant of antibody-binding specificity (Fig. 5ci). Together, this synthetic DNA fragment UMI library design facilitates quantification of the relative amplification of each unique DNA template between methods. The sUMI-seq PCR was applied to the synthetic DNA fragment library using either 5, 10, or 20 PCR cycles in PCR1, to test the effect of different PCR cycles, followed by PCR2 (20 cycles). In addition, a standard non-barcoded PCR using standard non-barcoded primers (i.e containing the gene-specific annealing region only), using the same synthetic DNA-UMI as template, was amplified with an equivalent approach (see methods). Each reaction condition successfully generated PCR amplicons that were subsequently sequenced by MiSeq (sequencing information in Table S2).

Firstly, we quantified the frequency of further amplification of PCR1 self-annealing MALBAC loops products depending on the number of PCR cycles. This is achieved through the assessment of the frequencies of the synthetic DNA-UMI per primer barcode pair (i.e. 1000*(“number of identical synthetic DNA-UMIs”/“number of sUMI-seq primer barcode pairs”), (Fig. 2b). As expected, the frequency of duplicated synthetic DNA-UMIs per sUMI-seq primer barcode pair was low (mean rate per sample of 0.392–1.335 per 1000 reads). The rate of duplicated synthetic DNA-UMIs per sUMI-seq primer barcode pair increased with the number of PCR 1 cycles that appeared to asymptote at 1.291 (Fig. 2b, 75% confidence intervals 0.939–2.536, p-value = 0.0168). This demonstrated only a low level of further amplification of the MALBAC loop amplicon in PCR1, and this level can be tailored depending on the number of PCR cycles. Importantly, between 5–10 cycles and between 10–20 cycles, less that a 2-fold increase in MALBAC-loop-specific amplification was observed. This suggests that the PCR1 step MALBAC-loop-specific amplification is non-exponential, and the PCR 1 step predominantly amplifies the original DNA template.

Next, we quantified and compared the relative amplification biases between sUMI-seq and standard non-barcoded PCR through synthetic DNA-UMIs amplification. To determine the effectiveness of the sUMI-seq primers in reducing the amplification biases, we also compared the relative amplification biases after filtering using or ignoring the sUMI-seq barcode information (Fig. 2ci). To account for differences in read depths between the different methods, each filtered dataset was subsampled to the same read depth across all samples (3000 reads), and relative amplification biases were calculated, defined as the maximum number of reads containing the same synthetic DNA-UMI. The mean relative amplification biases was calculated from 500 repeats per sample. (Fig. 2cii). Indeed, the mean relative biases were equivalent between sUMI-seq PCR ignoring the barcode information and the standard non-barcoded PCR. However, the mean relative biases were significantly lower in the sUMI-seq PCR using the barcode information compared to ignoring the barcode information (p-value=0.005). Together, this highlights the need for accounting for amplification biases.

**Figure 3:**
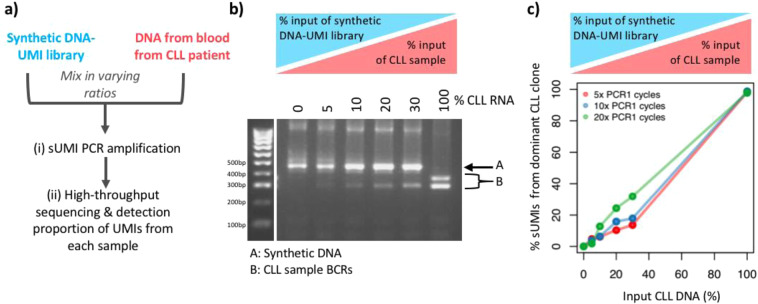
Titration of DNA pools using sUMI-seq. PCR (**a**) Schematic of titration experiment: To test the quantitative nature of sUMI-seq, a dilution series was performed of a peripheral blood (PB) DNA sample, from a chronic lymphocytic leukaemia (CLL) patient, mixed with the synthetic barcoded BCR DNA fragment library at varying ratios. sUMI-seq PCR was applied, again using either 5, 10, or 20 cycles in PCR1. (**b**) DNA agarose gel showing the BCR amplification of a titration of a CLL patient PB DNA sample into synthetic barcoded BCR fragments using 5 cycles in PCR1 and (**c)** the corresponding linear correlation between CLL DNA patient input and proportion of CLL BCR sequencing reads after accounting for primer barcodes based on PCR1 cycles.

Finally, a quantitative amplicon barcoding method should have a linear correlation between DNA template input and sequence output. To test this, we performed a dilution series of a peripheral blood (PB) DNA sample mixed with the synthetic UMI-DNA library at varying ratios, and sUMI-seq PCR was applied, again using either 5, 10, or 20 PCR1 cycles (Fig. 3a). The PB DNA sample was from a chronic lymphocytic leukaemia (CLL) patient, characterised by a clonal expansion of a single B cell clone, where >50% of all peripheral B cells contain a single IgH VDJ rearrangement (IGHV1–69*14-IGHJ6*02), as previously published^5^. Indeed, there was a strong linear relationship between CLL DNA input and proportion of sequencing reads after accounting for barcodes (Figs. 3b,c, S4). This suggests that sUMI-seq primers can be used to accurately correct amplification bias.

**Figure 4:**
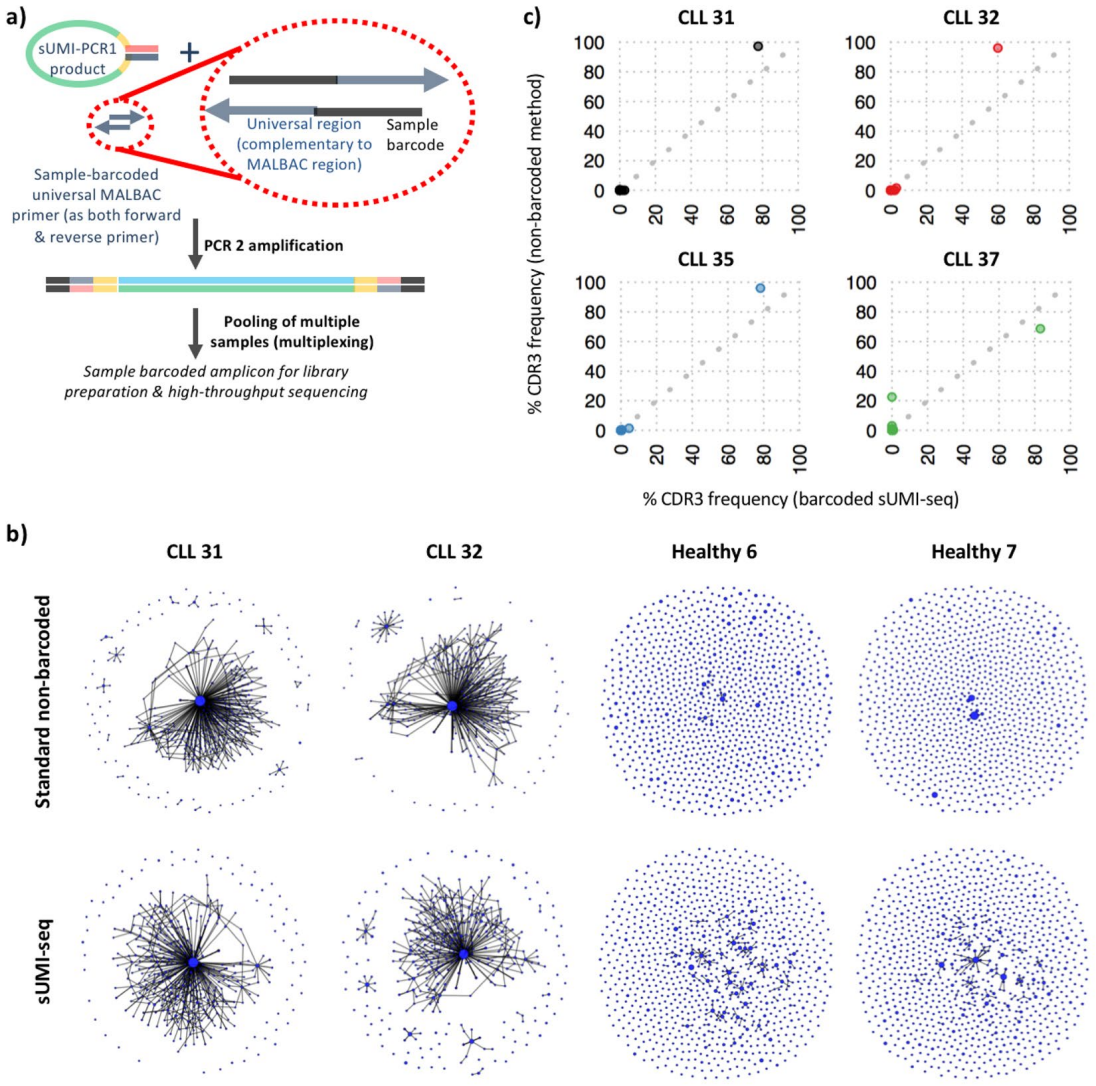
BCR repertoire sequencing by sUMI-seq. PCR (**a**) Schematic diagram of multiplex (sample-barcoded) sUMI-seq. used in PCR2 (**b)** Representative network plots of 2 chronic lymphocytic leukaemia (CLL) and 2 healthy individual BCR repertoires derived from PBMC DNA amplified by (top) the standard non-barcoded BCR amplification approach and (bottom) by sUMI-seq. Each vertex represents a unique BCR sequence (B cell clone), where relative vertex size is proportional to the number of identical BCR reads. Edges join vertices that differ by single nucleotide non-indel differences and clusters are collections of related, connected vertices. Networks are comprised of a subsample of 500 BCRs per sample. (**c**) Correlation of the frequencies of each B cell clone (defined by the CDR3 sequence) within the CLL patient samples derived from the sUMI-seq method versus the standard PCR method. The grey dotted line corresponds to y = x, and each point corresponds to a different CDR3 (B cell clone) sequence frequency.

We next applied sUMI-seq PCR to BCR sequencing of clinical samples, namely on a well-characterized cohort of peripheral blood mononuclear cell (PBMC) samples from 11 healthy individuals and 4 chronic lymphocytic leukemia (CLL) patients who have previously been sequenced using the conventional BCR non-barcoded amplification method^14^. Subsequent linearization of the amplicons in PCR2 with the inclusion of a sample-specific barcode in the linearization primers was used to facilitate efficient sample pooling before library preparation and high-throughput sequencing (Fig. 4a). This yielded between 4228–29372 unique sUMI-barcodes per sample after filtering for BCRs, comprising between 1055–4688 unique IgH V-D-J rearrangement per sample (Table S1). As previously observed, the healthy individual samples yielded diverse BCR repertoires (Figs. 4b and S6), whereas the CLL samples were characterized by the clonal expansion of a single malignant B cell clone (Fig. 4b), demonstrated by the increased maximum clone size and clonal diversification indices. The BCR sequences of dominant malignant BCR clones identified by sUMI-seq were identical to that of conventional BCR non-barcoded amplification methods and BCR amplification by RNA as previously published^5^ (Fig. S7). Furthermore, the frequency of each B cell clone, as defined by the CDR3 of the BCR sequence, was highly correlated with that of the conventional DNA amplification method (Fig. 4c). Together, this demonstrated that sUMI-seq PCR could be used to efficiently capture BCR repertoire data from DNA sources.

We next determined whether the capture of BCR repertoires were significantly improved using sUMI-seq. Given that sUMI-seq benefits from both error-correction and amplification bias-correction (see methods), we hypothesized that the estimation of (1) clonal diversity, (2) the level of somatic hypermutation and (3) mean amplicon length would be improved compared to standard non-barcoded BCR PCR amplification methods.

**Figure 5:**
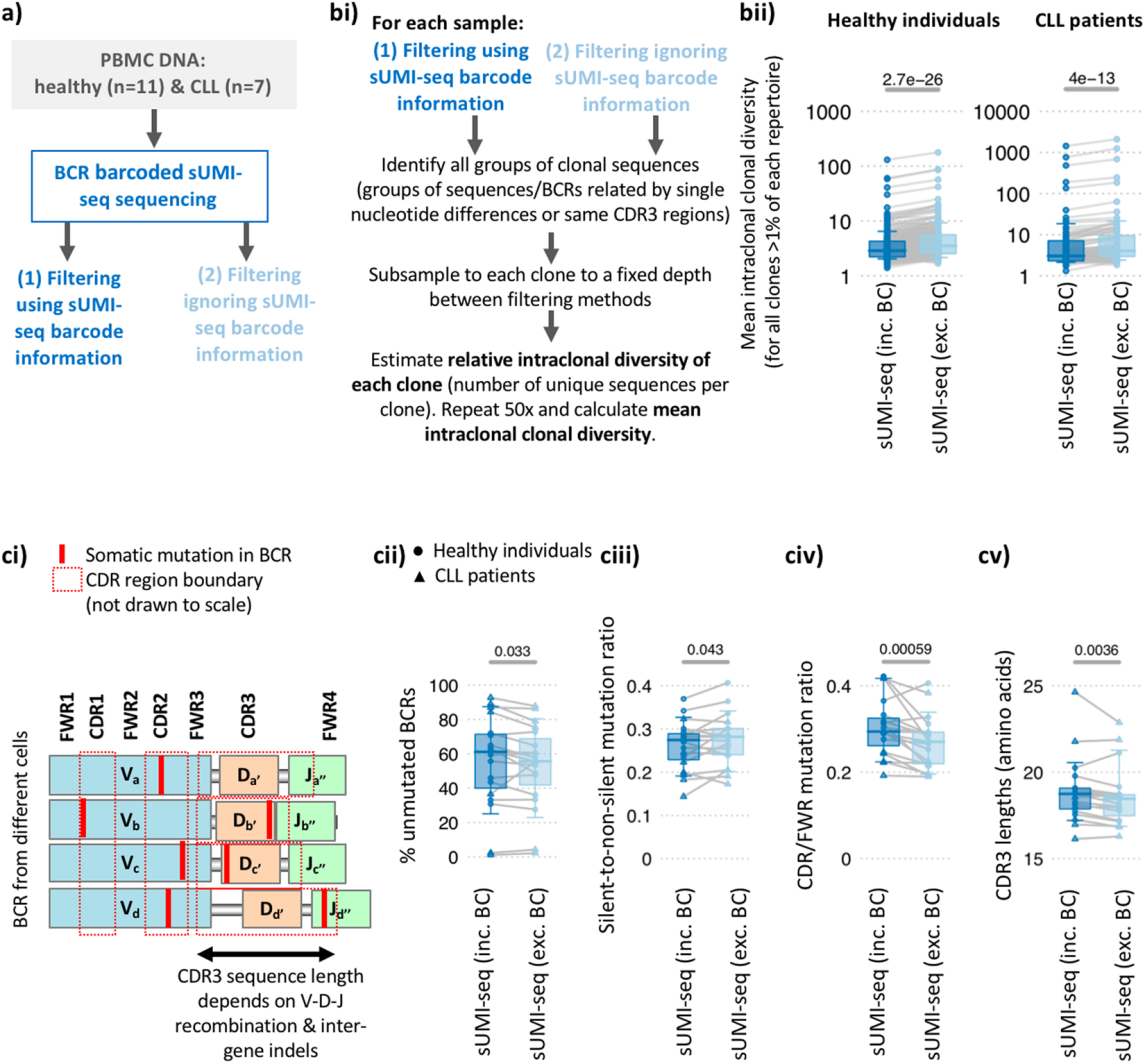
Comparison of BCR repertoire features between sUMI-seq PCR, filtering with and without barcode information (**a**) Schematic diagram of the comparison between sUMI-seq (filtering using barcode information) and sUMI-seq (filtering ignoring barcode information). (**bi)** Quantifying the mean intraclonal diversity: the relative intraclonal diversity of all clones >1% of the total repertoire in each sample was compared between the two methods. To account for the differences in read depth, the BCRs from each clone were subsampled to a fixed depth between filtering methods (0.75x the minimum number of sequences per clone across methods). The relative intraclonal diversity of each clone was defined as number of unique BCR sequences per clone after subsampling, and the mean intraclonal relative intraclonal diversity was determined through calculating the mean of 50 repeats. (**ii)** Boxplots of the mean intraclonal diversity between methods for all healthy (left) and CLL (right) patient samples, plotted on a logarithmic scale. Grey lines connect the mean relative clonal diversity measurements between methods. (**ci)** The schematic demonstrates the relative locations of the CDRs and FWRs within the BCR sequences (IgH VDJ). Boxplots quantify the differences in commonly used BCR repertoire features between methods including (**cii)** the proportion of unmutated BCRs (i.e. BCRs with no somatic hypermutations), (**ciii)** the ratio of silent-to-non-silent mutations, (**civ)** the CDR/FWR mutation ratio and (**cv)** CDR3 lengths (amino acids). The y axis provides the mean value per sample for each BCR repertoire feature, and grey lines the measurements between methods. Wilcoxon tests were performed in *R*.

Firstly, the relative clonal diversity of all clones representing>1% of the total repertoire in each sample was compared between filtering using sUMI-seq barcode information to correct for amplification biases and filtering ignoring the sUMI-seq barcode information, whilst accounting for read depth (Fig. 5a,bi). Indeed, the use of the sUMI-seq barcode information resulted in a significant reduction in estimated clonal diversity in all clones tested in both healthy (diverse) and CLL (clonal) BCR repertoires (p-values < 1e-10, Fig. 5bii). This suggests that standard PCR amplification methods overestimate the diversity of DNA pools due to the introduction of PCR amplification and sequencing errors, which can be corrected through the use of sUMI-seq primer barcoding. Secondly, the estimation of the level of somatic hypermutation (SHM) was significantly reduced when the sUMI-seq barcode information was used for filtering. This was demonstrated by a significantly higher proportion of unmutated BCR sequences (i.e. the IGHV region within 1 bp difference from the closest germline reference gene) when using the sUMI-seq primer barcoding (Fig. 5cii). The nature of the mutations, often reported in BCR sequencing studies^15^, was also significantly different when using error and amplification bias correction. This was shown both in terms of the lower silent-to-non-silent mutation ratio (p-value = 0.033, Fig. 5biii) and the locations of the mutations: namely a higher proportion of mutations occurring in the CDRs compared to the FWRs (p-value = 0.00059, Fig. 5civ). The latter is in agreement with previous studies where mutations are known to preferentially occur in the CDRs compared to the FWRs^16,17^.

Furthermore, the PCR amplification is known to preferentially amplify shorter amplicons. The CDR3 is the most variable region of the BCR sequence, driven in part by the combinations of different IGHV-D-J regions that are recombined during B cell maturation (Fig. 5ci**)**. Indeed, longer CDR3 lengths (longer than ~20 amino acids) are associated with both auto- and poly-reactivity and are often interrogated in BCR repertoire studies^18^. The mean CDR3 region length can be determined from the BCR sequencing data, and, indeed, significantly increased mean CDR3 lengths were observed using amplification-bias-correction via sUMI-seq compared to when error correction was not used (Fig. 5cv).

Together, this data suggests that the sUMI-seq barcoded approach represents a closer representation of the “ground truth” of the BCR repertoire compared to the non-barcoded repertoires. This demonstrates that the estimation of diversity, mutation and amplicon lengths of a mixed DNA pool are all significantly improved by sUMI-seq compared to conventional non-barcoded methods.

In summary, the sUMI-seq strategy allows for ultrasensitive barcoding of PCR amplicons from DNA for high-throughput sequencing, benefiting from significantly reduced PCR and sequencing errors and amplification biases leading to more accurate characterization of mixed DNA samples. We applied this method to immune receptor repertoire (BCR) profiling, where sUMI-seq captured both highly diverse and highly clonal B cell repertoires from healthy and CLL patients, respectively. sUMI-seq allowed for more accurate estimation of diversity, mutation and amplicon lengths, which are key analyses in many studies of mixed DNA variant pools. sUMI-seq can be easily applied to any PCR amplicons, and benefits from simplicity of primer design, straightforwardness of the amplification protocol with few steps, and streamlined method for incorporating sample barcodes in the second PCR. We have demonstrated the utility and power of this method in the characterization of complex immune receptor repertoire profiles, and this may be applied to a wide range of other applications in which characterizing DNA variants may be obscured by amplification bias or sequence error.

## Materials and Methods

### Samples

Peripheral blood mononuclear cells (PBMCs) were isolated from 10 mL of whole blood from healthy volunteers and CLL patients using Ficoll gradients (GE Healthcare). Total RNA was isolated using TRIzol and purified using RNeasy Mini Kit (Qiagen), including on-column DNase digestion according to the manufacturer’s instructions. Ethical approval for this study was obtained from the Eastern NHS Multi Research Ethics Committee (07/MRE05/44). Informed consent was obtained from all subjects enrolled and all experiments were performed in accordance with relevant guidelines and regulations.

### Design and amplification with barcoded primers

Gene specific sUMI-seq primers were designed according to Fig. S8.

#### sUMI-seq PCR 1 amplification with barcoded MALBAC primers

PCR1 was performed using 15 μL KAPA buffer (2×) (KAPA HIFI Hotstart PCR kit, Kapa Biosystems), 1 μL MALBAC IgH V (FR3) forward primer mix (10 µM) (containing 7 family specific primers designed to target the FR3 regions of VH1 through VH7 variable gene families) and 1 μL MALBAC reverse primer JH_(10 µM) (consensus sequence), 8 μL nuclease-free water, 5 μL DNA template (20 ng/μL), in a final volume of 30 μL. sUMI-seq primer sequences that amplify the BCR repertoire are provided in Table S1. The synthetic DNA library (UMI_DNA) was designed to be amplified with the same primer sets. The thermal cycling conditions for sUMI-seq PCR 1 were as follows: 1 cycle (95 °C–5 min); 5 cycles (98 °C–5 sec; 72 °C–2 min); 5 cycles (65 °C–10 sec, 72 °C–2 min); 5, 10, 20, or 30 cycles (98 °C–20 sec, 60 °C–1 min, 72 °C–2 min); 1 step (72 °C–10 min). PCR1 amplicons were then cleaned-up using 0.8x Agencourt AMPure XP beads (Beckman Coulter).

#### sUMI-seq PCR 2 amplification (without sample IDs)

The PCR2 reaction was performed using 17.5 μL of KAPA buffer (2×) (KAPA HIFI Hotstart PCR kit, Kapa Biosystems), 1 μL of 10 μM IgH V (FR3) forward primer mix and 1 μL of 10 μM MALBAC_UNI primers, 5.5 μL of nuclease-free water, 10 μL of DNA template (from PCR1), in a final volume of 35 μL. The thermal cycling conditions were as follows: 1 cycle (95 °C–5 min); 5 cycles (98 °C–5 sec; 72 °C–2 min); 5 cycles (65 °C–10 sec, 72 °C–2 min); 20 cycles (98 °C–20 sec, 60 °C–1 min, 72 °C–2 min); 1 step (72 °C–10 min).

#### sUMI-seq PCR 2 amplification (with sample barcode IDs)

The PCR2 reaction was performed using 17.5 μL KAPA buffer (2×) (KAPA HIFI Hotstart PCR kit, Kapa Biosystems), 1 μL MALBAC IgH V (FR3) forward primer mix (10 μM), and 1 μL MALBAC_UNI_Ind primer (10 μM) (choice of 1–12 barcodes) (Table S1), 5.5 μL nuclease-free water, 10 μL DNA template (from PCR1), for a total volume of 35 μL. The thermal cycling conditions were as follows: 1 cycle (95 °C–5 min); 5 cycles (98 °C–5 sec; 72 °C–2 min); 5 cycles (65 °C–10 sec, 72 °C–2 min); 20 cycles (98 °C–20 sec, 60 °C–1 min, 72 °C–2 min); 1 step (72 °C–10 min).

### Standard non-barcoded PCR amplification

This was performed using 15 μL KAPA buffer (2×) (KAPA HIFI Hotstart PCR kit, Kapa Biosystems), 1 μL IgH V (FR3) forward primer mix (10 μM) (standard non-barcoded primers), and 1μL reverse IgH-J (10 μM) (standard primers), 8 μL nuclease-free water, 5 μL DNA template (20 ng/μL), for a total volume of 30 μL. The thermal cycling conditions were as follows: 1 cycle (95 °C–5 min); 5 cycles (98 °C–5 sec; 72 °C–2 min); 5 cycles (65 °C–10 sec, 72 °C–2 min); 5, 10, or 20 cycles (98 °C–20 sec, 60 °C–1 min, 72 °C–2 min); 1 step (72 °C–10 min).

### High-throughput sequencing and QC

PCR2 DNA amplicons were cleaned-up using 0.8x Agencourt AMPure XP beads (bead-based size selection) and checked using electrophoresis on a 2% agarose gel. MiSeq libraries were prepared using KAPA protocols (KK8722 and KK8504) and sequenced using 300 bp pair-end MiSeq (Illumina). Raw MiSeq reads were filtered for base quality (median Phred score >32) using QUASR (http://sourceforge.net/projects/quasr/)^3^.

MiSeq forward and reverse reads were merged together if they contained an identical overlapping region of >50 bp, or otherwise discarded.

### For the sUMI-seq filtering pipeline

Universal barcoded regions were identified in reads and orientated to read from forward (IgH V)-primer to reverse (IgH-J) region primer. The barcoded region within each primer was identified and checked for conserved bases. **Error-correction and amplification bias correction:** Groups of sequencing reads containing the same sUMI-seq primer UMIs originate from the same DNA template, and therefore a consensus sequence was generated from these groups. This reduces amplification biases (i.e. the effect of differential amplification of DNA templates), as well as correcting potential PCR/sequencing errors. Consensus sequences were retained only if there was a per-base agreement of 80% between all sequencing reads containing the same UMI. For groups of 4 of fewer sequencing reads containing the same UMI, there needed to be complete agreement between sequences after alignment, otherwise were discarded. This is summarised in Fig. S5.

### For the standard filtering pipeline

The primer regions within the sequencing reads were determined. All sequences without identifiable primer annealing regions were discarded.

### Quantifying the frequency of further amplification of that PCR1 self-annealing MALBAC loops products

For each sequence within the synthetic UMI-DNA datasets, the synthetic DNA-UMIs and primer barcode pairs were identified. From this, the proportion of sequences which contained DNA-UMIs associated with more than one primer barcode pairs was determined, and normalised to the total number of reads (provided as a rate per 1000 reads): $1000\ast (\frac{{\rm{number}}\,{\rm{of}}\,{\rm{identical}}\,{\rm{synthetic}}\,{\rm{DNA}}-{\rm{UMIs}}\ast {\rm{number}}\,{\rm{of}}\,{\rm{sUMI}}-{\rm{seq}}\,{\rm{primer}}\,{\rm{barcode}}\,{\rm{pairs}}}{{\rm{number}}\,{\rm{of}}\,{\rm{BC}}-{\rm{MALBAC}}\,{\rm{primer}}\,{\rm{pairs}}/{\rm{number}}\,{\rm{of}}\,{\rm{reads}}})$

### Quantifying amplification bias using the synthetic DNA-UMIs library and comparing sUMI PCR to standard PCR amplification

The relative amplification biases were compared between (1) the sUMI-seq method using sUMI-seq barcode information (using the sUMI-seq filtering pipeline), (2) the sUMI-seq method ignoring the sUMI-seq barcode information (using the standard filtering pipeline), and (3) using the standard non-barcoded PCR amplification method and the standard filtering pipeline. To account for differences in read depths between the different methods, each filtered dataset was subsampled to the same read depth across all samples (3000 reads), defined as the maximum number of reads containing the same synthetic DNA-UMI:

$$amplification\,bias\,(per\,subsample)\,=\,{\max }({n}_{i})$$

where *n*_*i*_ is number of reads containing synthetic DNA − UMI *i*. The mean relative amplification biases was calculated from 500 repeats per sample. Wilcoxon tests were performed in *R*.

### BCR sequence filtering

Sequences without complete reading frames and non-immunoglobulin sequences were removed and only reads with significant similarity to reference IgH variable genes (V-D-J) from the IMGT database were retained using BLAST^19^. Sequence annotation, including somatic hypermutation, CDR3 regions and IGHV gene usages, were defined via IMGT V-QUEST, where repertoire differences were performed by custom scripts in Python, and statistics were performed in *R* using Wilcoxon tests for significance.

### BCR repertoire generation and network analysis

The network generation algorithm and network properties were calculated as in Bashford-Rogers *et al*.^5^: each vertex represents a unique sequence, where relative vertex size is proportional to the number of identical reads. Edges join vertices that differ by single nucleotide non-indel differences and clusters are collections of related, connected vertices.

A **clone (cluster)** refers to clonally-related B cells, containing BCRs with identical CDR3 regions and IgH gene usage, or differing by single point mutations, such as through somatic hypermutation.

**Clonality diversity** refers to the relative number of clonally-related, but distinct, B cells within a clone. In the context of BCR sequencing, this is a measure of the number of unique clonally-related BCRs (clone members). Sequence repertoire parameters that were dependent on sequencing depth were generated by subsampling each sequencing sample to a specified clone depth. This includes the Clonal Diversification index, was measured by cluster Renyi Index as defined in Bashford-Rogers *et al*.^5^. This is calculated from the distribution of the number of unique VDJ region sequences per clone within subsampled BCR repertoires at specified depth of 1000 clones. The mean of 100 repeats of resulting Clonal Diversification indices was determined. Clone size distributions were also calculated from the same subsamples and a mean of 100 repeats was determined.

### BCR network sampling to preserve the overall clonal structure of visual representation

To obtain representative subgraph of a network that preserves the overall relative clonal architecture whilst providing visual representations that distinguish between samples of different clonalities, clone subsampling was used as described in^15^. One thousand clones are subsampled and a network generated from all BCRs from these clones. Subsampling was performed 100 times, and the sample that contained a maximum clone size closest to the median of all subsamples greater than the unsampled maximum clone size was chosen.

### Ethics approval and consent to participate

Ethical approval for this study was obtained from the Eastern NHS Multi Research Ethics Committee (07/MRE05/44). Informed consent was obtained from all subjects enrolled.

## Supplementary information

Supplementary Information.

## Data Availability

Code is made available at https://github.com/rbr1/sUMI_processing_pipeline. The datasets used and/or analysed during the current study are available from the corresponding author on reasonable request. All sequencing data will be uploaded to the EGA.
