# Author Correction: Ultrasensitive amplicon barcoding for next-generation sequencing facilitating sequence error and amplification-bias correction

**DOI:** 10.1038/s41598-020-74321-4

**Published:** 2020-10-07

**Authors:** Ibrahim Ahmed, Felicia A. Tucci, Aure Aflalo, Kenneth G. C. Smith, Rachael J. M. Bashford-Rogers

**Affiliations:** 1 Department of Medicine, University of Cambridge, Cambridge, United Kingdom; 2 Present Address: Faculty of Biology, Medicine and Health, University of Manchester, Michael Smith Building, Oxford Road, Manchester, M13 9PT UK; 3 Wellcome Centre for Human Genetics, University of Oxford, Oxford, United Kingdom; 4 Department of Pathology, University of Cambridge, Cambridge, United Kingdom; 5 Cambridge Institute of Therapeutic Immunology and Infectious Disease, Jeffrey Cheah Biomedical Centre, University of Cambridge, Cambridge, United Kingdom

Correction to: *Scientific Reports* https://doi.org/10.1038/s41598-020-67290-1, published online 29 June 2020

A supplementary file containing Supplementary Figures S1-S8 was omitted from this Article. This file is linked to this correction notice.

## Supplementary information

Supplementary Information

