# Supplementary material for: Author Correction: Ultrasensitive amplicon barcoding for next-generation sequencing facilitating sequence error and amplification-bias correction
Source: Sci Rep. 2020 Oct 7;10:17010. doi: 10.1038/s41598-020-74321-4 (PMC7538963; doi:10.1038/s41598-020-74321-4)
Supplement: Supplementary file 1 — Supplementary Information [file 41598_2020_74321_MOESM1_ESM.pdf]

Ultrasensitive amplicon barcoding for next-generation sequencing facilitating sequence error and amplification-bias correction

Ibrahim Ahmed[1,2], Felicia A. Tucci[3], Aure Aflalo[1,4], Kenneth G. C. Smith[1,5], Rachael Bashford-Rogers[1,3,*]

[1] Department of Medicine, University of Cambridge, Cambridge, United Kingdom.
[2] Current address: Faculty of Biology, Medicine and Health, University of Manchester, Michael Smith Building, Oxford Road, Manchester M13 9PT, UK
[3] Wellcome Centre for Human Genetics, University of Oxford, Oxford, United Kingdom.
[4] Department of Pathology, University of Cambridge, Cambridge, United Kingdom
[5] Cambridge Institute of Therapeutic Immunology and Infectious Disease, Jeffrey Cheah Biomedical Centre, University of Cambridge, Cambridge, UK.

***Corresponding author:** rbr1@well.ox.ac.uk

Keywords
Next generation sequencing
BCR repertoire
Error correction
Amplification bias
Amplicon sequencing

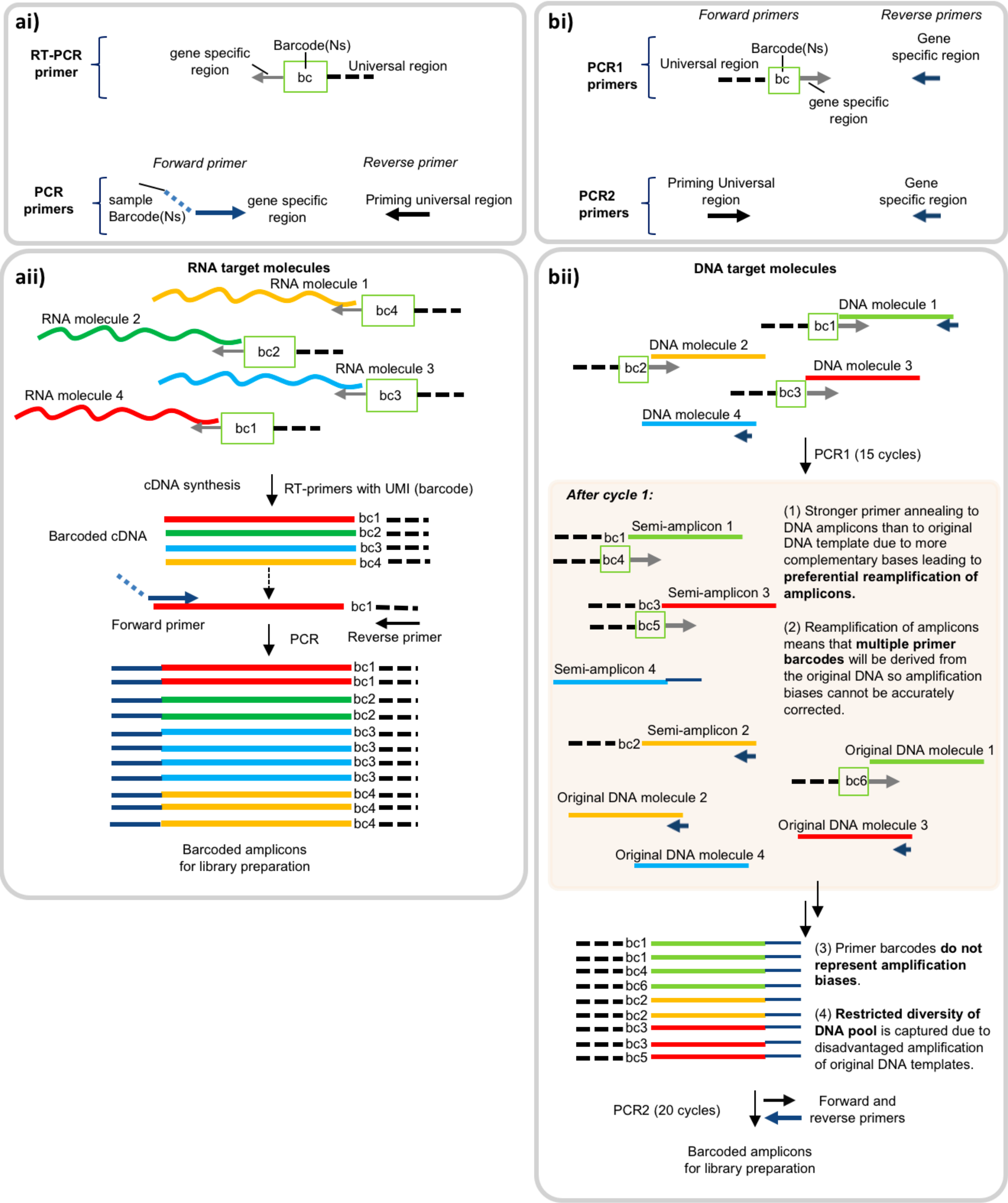

Figure S1. Schematic diagrams of PCR amplification methods. a) Schematic diagram of the barcoded RT-PCR **i)** primers and **ii)** amplification reaction. In the reverse transcription (RT) step, the primer anneals to the constant region of the BCR mRNA to generate cDNA with a random 12-nucleotide barcode. The product is cleaned and PCR-amplified using multiple primers that bind to the FR1 region of the IgH genes along with a universal sequence complementary to the end of the reverse-transcription primer. **b)** Schematic diagram of the non-sUMI barcoded **i)** primers and **ii)** PCR amplification reaction. Non-sUMI barcoded amplification uses primers with barcodes adjacent to the gene-specific regions. Standard PCR amplification is performed, where the amplicons generated have stronger binding to primers due to longer complementary region. This means that the primer barcodes do not represent amplification biases and also results in a restricted diversity of DNA pool is captured due to disadvantaged amplification of original DNA templates and a skewed amplicon library.

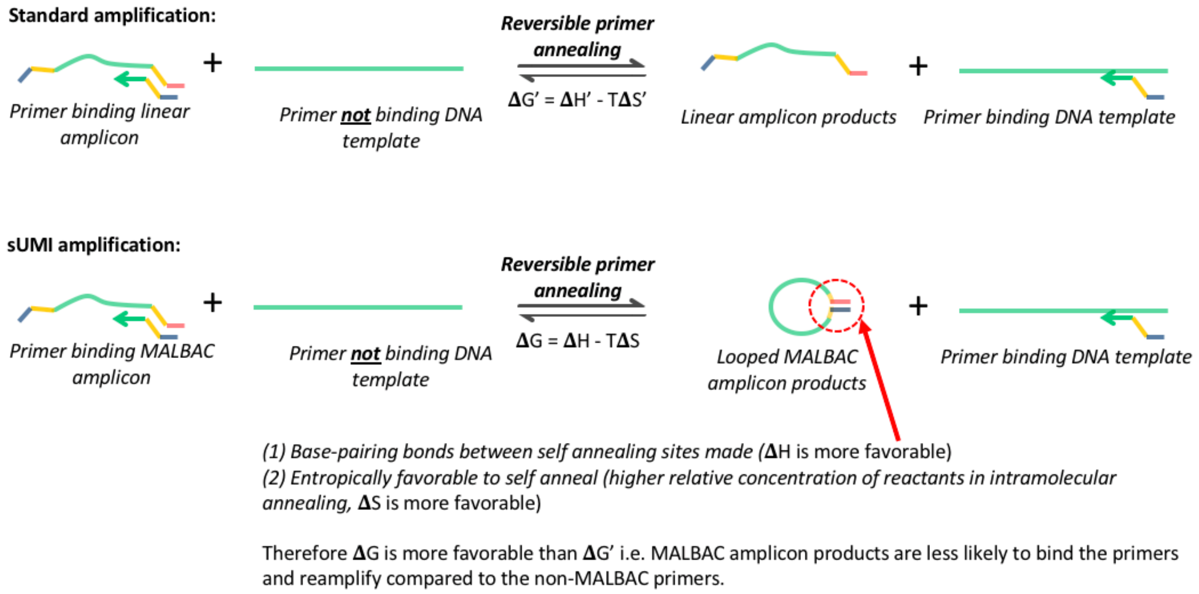

Figure S2. MALBAC amplicons (in PCR 1 of sUMI-seq) preferentially do not further amplify due to the thermodynamic and kinetic preference for loop closure compared to further primer annealing. Primer binding can be considered a reversible reaction between the amplicons and the original DNA template. The Gibbs free energy change, ΔG, is the minimum thermodynamic work needed to drive a reaction, and is defined as the sum of the enthalpy change (ΔH) and the entropy change (ΔS) multiplied by the negative of temperature (-T). Here, ΔG' and ΔG refer to the Gibbs free energy change between the primer binding to the PCR amplicon (left side) and the original DNA template (right side) for the standard PCR and sUMI-seq PCR 1 amplification respectively. The MALBAC amplicons are less likely to bind the primers and reamplify compared to the standard (non-MALBAC) amplicons due to a more favorable ΔG (i.e. ΔG< ΔG') resulting from: (1) base-pairing bonds between self-annealing sites are made upon loop formation (ΔH is more favorable) and (2) it is entropically favorable to self-anneal due to a higher relative concentration of reactants in intramolecular annealing (ΔS is more favorable). Therefore, the MALBAC amplicon loops preferentially do not further amplify.

a)

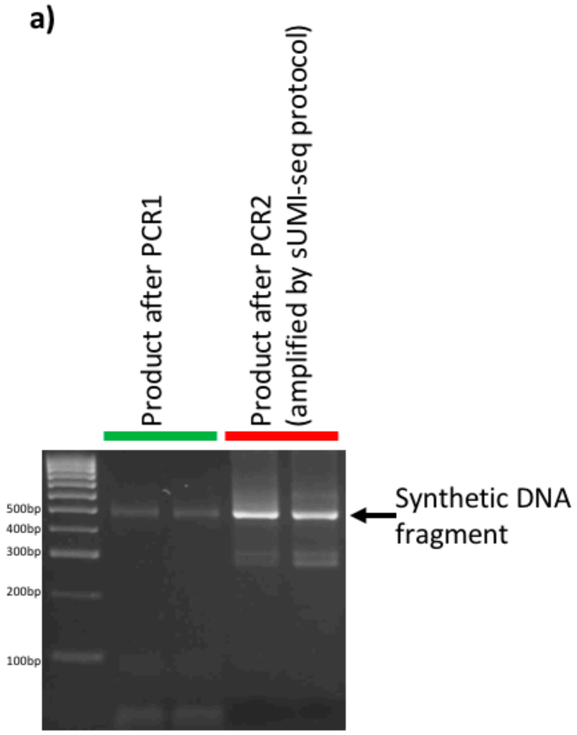

b)

```
Forward       ----------------------------------------aagttccagggcagagtcac
Reverse_RC    ---ttttgatcctgaagatggtgaaacaatctacgcacagaagttccagggcgaagtcac
Template      ---ttttgatcctgaagatggtgaaacaatctacgcacagaagttccagggcagagtcac
                 *********************************************************

Forward       catgaccgaggacacatctacagtacacagcctacatggagctga-gcagcctgagatct
Reverse_RC    catgaccgaggacacatctacag-acacagcctacatggagctgaggcagcctgagatct
Template      catgaccgaggacacatctacag-acacagcctacatggagctga-gcagcctgagatct
              *********************** ********************* **************

Forward       gaggacacagccatgtattactgtgcaagggagtgcatccgccccaacccttttccccct
Reverse_RC    gaggacacagccatgtattactgtgcaagggagtgcatccgccccaacccttttccccct
Template      gaggacacagccatgtattactgtgcaagggagtgcatccgccccaacccttttccccct
              ************************************************************

Forward       cgtctcctgtgagannnncccgtcggatacgagcagcgtggccgttggctgcctcgcaca
Reverse_RC    cgtctcctgtgagaattccccgtcggatacgagcagcgtggccgttggctgcctcgcaca
Template      cgtctcctgtgagaattccccgtcggatacgagcagcgtggccgttggctgcctcgcaca
              **************    ******************************************

                         Barcode region
Forward       ggacttccttcnnnnnnnnnnnnnnnagtggcggcagtggctacgtcggggctggcgcct
Reverse_RC    ggacttccttc----nnnnnnnnnnnagtggcggcagtggctacgtcggggctggngcct
Template      ggacttccttcnnnnnnnnnnnnnnnagtggcggcagtggctacgtcggggctggcgcct
              ***********               ***************************** ****

Forward       acagctggttcgacccctggggcgagggaaccctgg
Reverse_RC    acagc-------------------------------
Template      acagctggttcgacccctggggccagggaaccctgg
              *********************** ************
```

Figure S3. Amplification of the synthetic DNA-UMI fragment library. a) DNA amplification of the synthetic DNA-UMI fragment library containing an internal DNA barcode unique to each DNA molecule. The agarose gel shows PCR products after PCR1 (lanes 2-3), after PCR2 using the sUMI-seq protocol (lanes 4,5). **b)** Alignment of the Sanger sequencing results of the sUMI-sec amplification of the synthetic DNA fragment with the template design. The barcoded region containing the internal DNA barcode unique to each DNA molecule is highlighted in red.

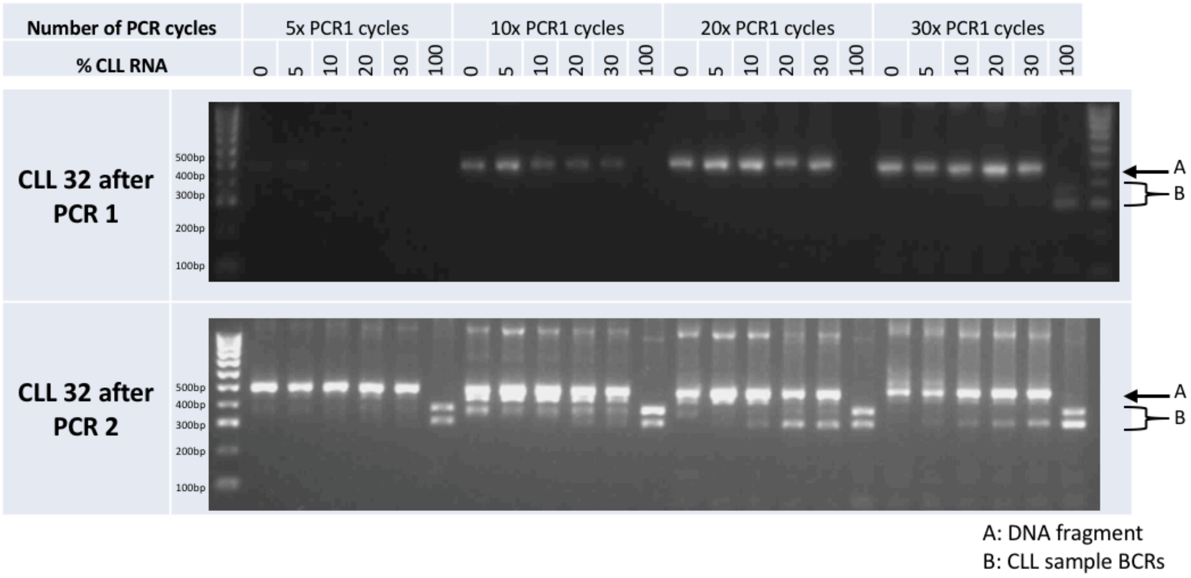

Figure S4. Agarose gels of the amplification of the dilution series of the DNA fragment and CLL samples for CLL 32 after **(top)** PCR1 and **(bottom)** PCR2.

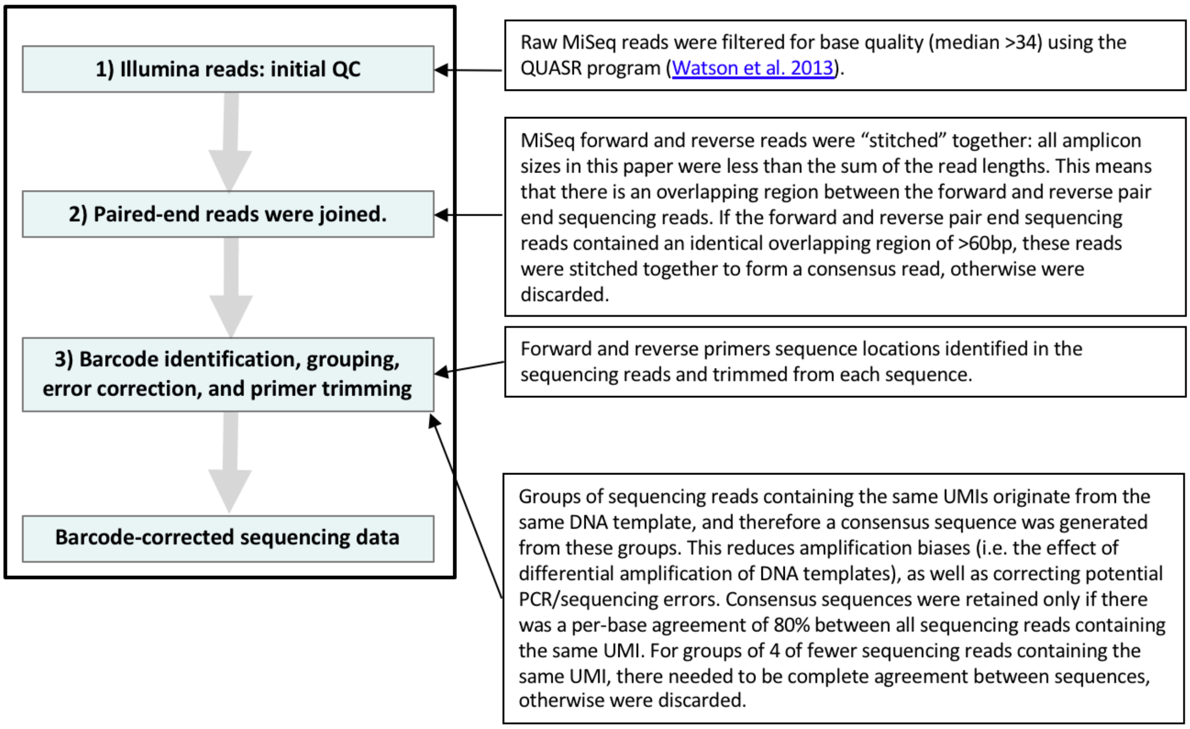

Figure S5. Outline of informatics pipeline.

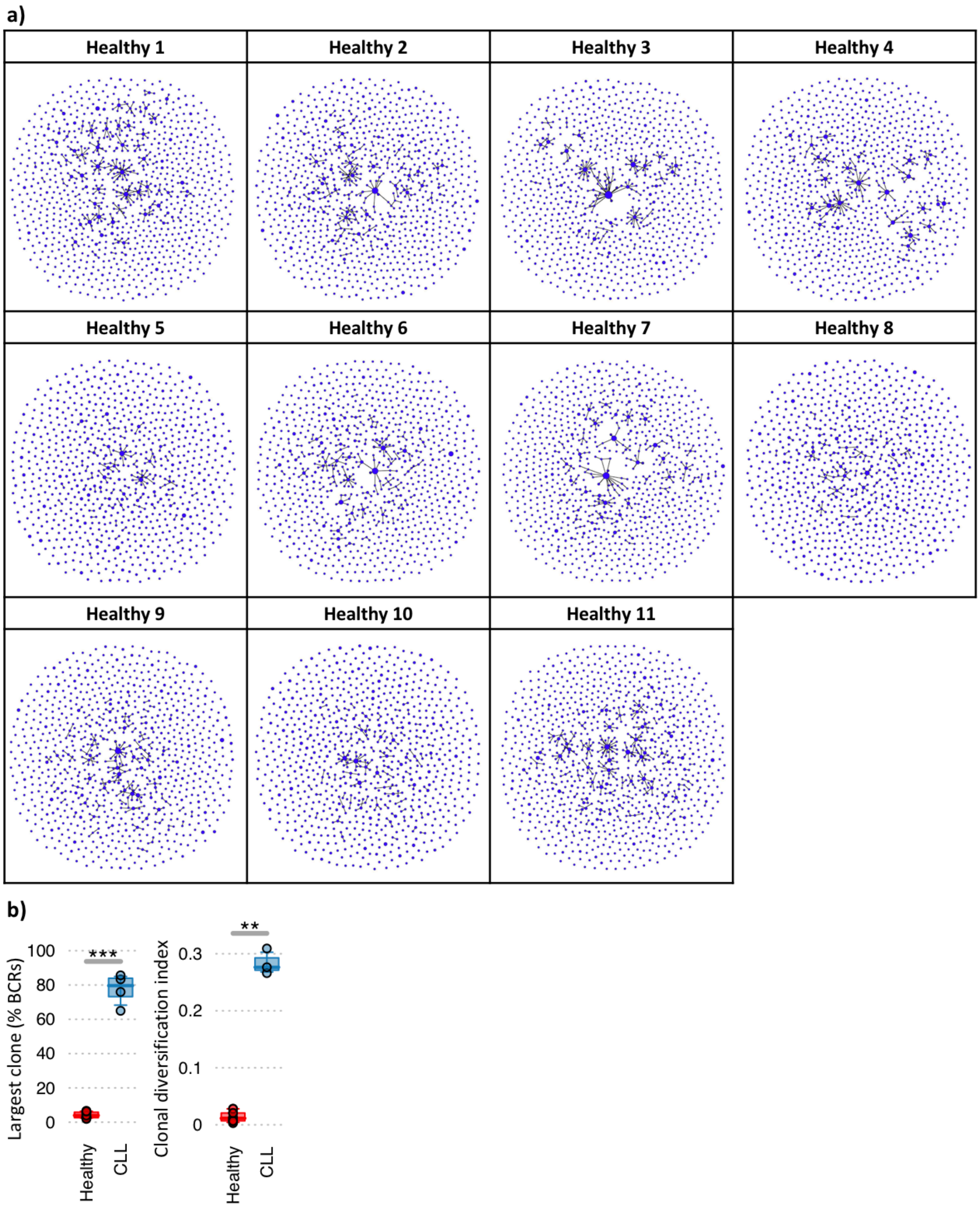

Figure S6. a) BCR networks for healthy PBMC samples. Each vertex represents a unique BCR sequence, where relative vertex size is proportional to the number of identical reads. Edges join vertices that differ by single nucleotide non-indel differences and clusters are collections of related, connected vertices. Networks are comprised of a subsample of 2000 clones. **b)** B cell receptor repertoire-derived metrics using the sUMI-seq method between healthy individuals and CLL patients. ** denotes p-values <0.005 and *** <0.0005, by T-test.

```
RNA_CLL_31       ------------------cgcctggctccagggaaggggctggagtgggtggccaacata
Stnd_DNA_CLL_31 tcatggatgacctgggtccgcctggctccagggaaggggctggagtgggtggccaacata
sUMI-seq_CLL_31   ------------------------------------------gagtgggtggccaacata
                                                           ******************

RNA_CLL_31       aaacaagatggaagtgaggaacactatgtggactctgtgaagggccgattcaccatctcc
Stnd_DNA_CLL_31 aaacaagatggaagtgaggaacactatgtggactctgtgaagggccgattcaccatctcc
sUMI-seq_CLL_31   aaacaagatggaagtgaggaacactatgtggactctgtgaagggccgattcaccatctcc
                ************************************************************

RNA_CLL_31       agagacaacgcgaagagttcactgtatctgcaaatgagcagcctgagagtcgaggatacg
Stnd_DNA_CLL_31 agagacaacgcgaagagttcactgtatctgcaaatgagcagcctgagagtcgaggatacg
sUMI-seq_CLL_31   agagacaacgcgaagagttcactgtatctgcaaatgagcagcctgagagtcgaggatacg
                ************************************************************

RNA_CLL_31       gctgtgtattactgtgcgagagatccatatagtgggaacttcggtgcttttgatatctgg
Stnd_DNA_CLL_31 gctgtgtattactgtgcgagagatccatatagtgggaacttcggtgcttttgatatctgg
sUMI-seq_CLL_31   gctgtgtattactgtgcgagagatccatatagtgggaacttcggtgcttttgatatctgg
                ************************************************************

RNA_CLL_31       ggccaagggacaga
Stnd_DNA_CLL_31 ggccaagggacagt
sUMI-seq_CLL_31   ggccaagggacagt
                *************

RNA_CLL_32       gcccctggacaagggcttgagtggatgggagggatcatccctatctttggtacagcaaac
Stnd_DNA_CLL_32 ---------caagggcttgagtggatgggagggatcatccctatctttggtacagcaaac
sUMI-seq_CLL_32   -------------------------------ggatcatccctatctttggtacagcaaac
                                               *****************************

RNA_CLL_32       tacgcacagaagttccagggcagagtcacgattaccgcggacaaatccacgagcacagcc
Stnd_DNA_CLL_32 tacgcacagaagttccagggcagagtcacgattaccgcggacaaatccacgagcacagcc
sUMI-seq_CLL_32   tacgcacagaagttccagggcagagtcacgattaccgcggacaaatccacgagcacagcc
                ************************************************************

RNA_CLL_32       tacatggagctgagcagcctgagatctgaggacacggccgtgtattactgtgcgagaggg
Stnd_DNA_CLL_32 tacatggagctgagcagcctgagatctgaggacacggccgtgtattactgtgcgagaggg
sUMI-seq_CLL_32   tacatggagctgagcagcctgagatctgaggacacggccgtgtattactgtgcgagaggg
                ************************************************************

RNA_CLL_32       cttccgaattacgattttggagtggttattctcccgcctactactactactacggtatg
Stnd_DNA_CLL_32 cttccgaattacgattttggagtggttattctcccgcctactactactactacggtatg
sUMI-seq_CLL_32   cttccgaattacgattttggagtggttattctcccgcctactactactactacggtatg
                ************************************************************

RNA_CLL_32       gacgtctggggccaagggaccag
Stnd_DNA_CLL_32 gacgtctggggccaagggaccac
sUMI-seq_CLL_32   gacgtctggggccaagggaccac
                **********************

RNA_CLL_35       ---------------------agtagttactactggagctggatccggcagcccccaggg
Stnd_DNA_CLL_35 actgtctctggtggctccatcagtagttactactggagctggatccggcagcccccaggg
sUMI-seq_CLL_35   ------------------------------------------------------------


RNA_CLL_35       aagggactggagtggattgggtatatctattacagtgggagcaccaactacaacccctcc
Stnd_DNA_CLL_35 aagggactggagtggattgggtatatctattacagtgggagcaccaactacaacccctcc
sUMI-seq_CLL_35   ----------agtggattgggtatatctattacagtgggagcaccaactacaacccctcc
                          **************************************************

RNA_CLL_35       ctcaagagtcgagtcaccatatcagtagacacgtccaagaaccagttctccctgaagctg
Stnd_DNA_CLL_35 ctcaagagtcgagtcaccatatcagtagacacgtccaagaaccagttctccctgaagctg
sUMI-seq_CLL_35   ctcaagagtcgagtcaccatatcagtagacacgtccaagaaccagttctccctgaagctg
                ************************************************************

RNA_CLL_35       agctctgtgaccgctgcggacacggccgtgtattactgtgcgagagataaaactggggat
Stnd_DNA_CLL_35 agctctgtgaccgctgcggacacggccgtgtattactgtgcgagagataaaactggggat
sUMI-seq_CLL_35   agctctgtgaccgctgcggacacggccgtgtattactgtgcgagagataaaactggggat
                ************************************************************

RNA_CLL_35       tactactactactactacatggacgtctggggcaaagggaccaa
Stnd_DNA_CLL_35 tactactactactactacatggacgtctggggcaaagggacca-
sUMI-seq_CLL_35   tactactactactactacatggacgtctggggcaaagggaccac
                *******************************************

RNA_CLL_37       ---------------------gcccctggacaaggacttgagtggatgggatggatccat
Stnd_DNA_CLL_37 gccataaattgggtgcgacaggcccctggacaaggacttgagtggatgggatggatccat
sUMI-seq_CLL_37   -------------------------------------ttgagtggatgggatggatccat
                                                     ***********************

RNA_CLL_37       accaaaactggcaacccaacgtatgcccagggcttcacaggacggtttgtcttctccttg
Stnd_DNA_CLL_37 accaaaactggcaacccaacgtatgcccagggcttcacaggacggtttgtcttctccttg
sUMI-seq_CLL_37   accaaaactggcaacccaacgtatgcccagggcttcacaggacggtttgtcttctccttg
                ************************************************************

RNA_CLL_37       ggcacctctgtcagcacggcatatctgcacatcaacagcctaagggctgaggacactgcc
Stnd_DNA_CLL_37 ggcacctctgtcagcacggcatatctgcacatcaacagcctaagggctgaggacactgcc
sUMI-seq_CLL_37   ggcacctctgtcagcacggcatatctgcacatcaacagcctaagggctgaggacactgcc
                ************************************************************

RNA_CLL_37       atgtattactgtgcgagaggcgtctattgtaatagtggcagttgttcaggggggccggcag
Stnd_DNA_CLL_37 atgtattactgtgcgagaggcgtctattgtaatagtggcagttgttcaggggggccggcag
sUMI-seq_CLL_37   atgtattactgtgcgagaggcgtctattgtaatagtggcagttgttcaggggggccggcag
                ************************************************************

RNA_CLL_37       cctttgataggtggggccaagggacaat
Stnd_DNA_CLL_37 cctttgataggtggggccaagggacaat
sUMI-seq_CLL_37   cctttgataggtggggccaagggacaat
                ****************************
```

Figure S7. Alignment of dominant malignant CLL BCR sequences using sUMI-seq, the standard non-barcoded amplification (denoted Stnd_DNA_CLL), and RNA sequencing (denoted RNA_CLL) for the 4 CLL samples.

PCR 1 primers:

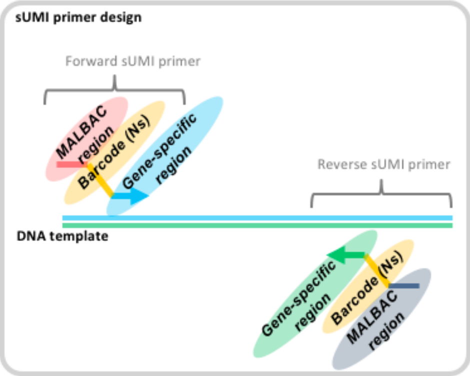

Forward sUMI primer(s):

MALBAC region	*Barcode (UMI) region*	*Gene-specific region*
GTGAGTGATGGTTGAGGTAGTGTGGAG	NNNNNNNN	TGGAGCTGAGCAGCCTGAGATCTGA
GTGAGTGATGGTTGAGGTAGTGTGGAG	NNNNNNNN	CAATGACCAACATGGACCCTGTGGA
GTGAGTGATGGTTGAGGTAGTGTGGAG	NNNNNNNN	TCTGCAAATGAACAGCCTGAGAGCC
GTGAGTGATGGTTGAGGTAGTGTGGAG	NNNNNNNN	GAGCTCTGTGACCGCCGCGGACACG
GTGAGTGATGGTTGAGGTAGTGTGGAG	NNNNNNNN	CAGCACCGCCTACCTGCAGTGGAGC
GTGAGTGATGGTTGAGGTAGTGTGGAG	NNNNNNNN	GTTCTCCCTGCAGCTGAACTCTGTG
GTGAGTGATGGTTGAGGTAGTGTGGAG	NNNNNNNN	CAGCACGGCATATCTGCAGATCAG

Reverse sUMI primer:

MALBAC region	*Barcode (UMI) region*	*Gene-specific region*
GTGAGTGATGGTTGAGGTAGTGTGGAG	NNNNNNNN	CTTACCTGAGGAGACGGTGACC

PCR 2 (linearization) primers:

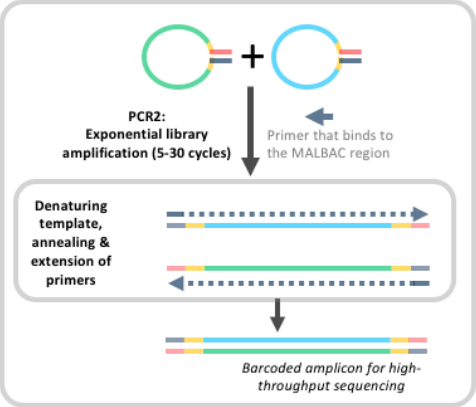

Forward and reverse linearization primer (without sample barcode):

Anneals to MALBAC region
GTGAGTGATGGTTGAGGTAGTGTGGAG

Forward and reverse linearization primer (with example sample barcode):

Sample barcode	*Anneals to MALBAC region*
Taggtatt	GTGAGTGATGGTTGAGGTAGTGTGGAG

Figure S8. Primer design strategy.
